# Editorial: Natural language processing and artificial intelligence tools to explore the relationship between language and schizophrenia from diagnosis to care

**DOI:** 10.3389/fpsyt.2025.1666275

**Published:** 2025-08-13

**Authors:** Olivier Dufor, Amir Hossein Nikzad, Valeria Lucarini, Christophe Lemey

**Affiliations:** ^1^ L@bISEN, ISEN Yncréa Oueste, Caen, France; ^2^ Institute of Behavioral Science, Feinstein Institutes for Medical Research, Northwell Health, Manhasset, NY, United States; ^3^ Zucker Hillside Hospital, New York, NY, United States; ^4^ Institute of Psychiatry and Neuroscience of Paris (IPNP), INSERM U1266, Team: Pathophysiology of Psychiatric Disorders: Development and Vulnerability, Université Paris Cité, Paris, France; ^5^ GHU Paris Psychiatrie et Neurosciences, CJAAD, Evaluation, Prevention and Therapeutic Innovation Department, Hôpital Sainte Anne, Paris, France; ^6^ CNRS GDR 3557-Institut de Psychiatrie, Paris, France; ^7^ URCI University Hospital, Department of Adult Psychiatry, Brest, France; ^8^ Sorbonne University, INSERM, Pierre-Louis Institute of Epidemiology and Public Health, Paris, France; ^9^ IMT Atlantique, Lab-STICC, Technopôle Brest-Iroise, Brest, France

**Keywords:** schizophrenia, natural language processing, linguistic markers, psychosis, early detection

Schizophrenia is a heterogeneous disorder classically defined by three symptom clusters: positive symptoms (such as hallucinations and delusions), negative symptoms (including affective flattening, avolition, and social withdrawal), and disorganization symptoms (notably thought disorder and incoherent speech). These manifest notably in language disturbances characterized by fragmented, disorganized, or impoverished speech, alongside motor and praxis difficulties as well as impaired social interactions, reflecting underlying neural circuit disruptions (1). Several studies have shown that speech behavior in particular, plays a central role in the symptomatology and diagnosis of patient outcomes (2, 3). A large body of clinical evidence suggests that early detection of schizophrenia spectrum disorder (SSD) and adequate care management and psychosocial interventions can help patients recover from the disease and integrate with the community (4, 5).

In this context, the evaluation of spoken language of patients with SSD or at risk of developing schizophrenia has repeatedly demonstrated its prognostic and diagnostic value (6, 7).

As a proxy for mental activity, language disorders, which represent most of the expression of “formal thought disorders”, manifest themselves through disorganization of speech, loss of coherence, and alteration of emotional expression (8). With natural language processing techniques (NLP), a whole collection of new features appears to contribute to the clinical picture of SSD and clinical high risk for psychosis (CHR-P). While semantic coherence emerge as the principal marker in many studies (9), others emphasized on emotional prosody (10). Nevertheless, most linguistic markers, when considered independently, lack of specificity for schizophrenia and symptomatic alterations go beyond the simple diagnostic framework of schizophrenia or psychosis (11). Then, a multimodal approach, integrating linguistic data with other clinical and biological markers, holds promises to enhance the accuracy and richness of detection and assessments of at risk patients (12, 13).

The objective of this topic was to bring together the most advanced studies in the field of diagnosing and predicting the future of patients with SSD and/or CHR-P from linguistic markers and to think about how they can be mixed in different pathological contexts and trajectories.

Among the articles that make up this topic, that of Just et al., highlights semantic incoherence, meaningly the inability to maintain a logical thread in the discourse. The authors demonstrate that semantic incoherence is one of the key elements in non-affective psychosis that could help in diagnosis. On top of negative symptoms scores which correlate with coherence independently of the embedding model, inpatient care, disorganized score and excitement score add up to the board when Word2Vec method is used.

In addition to the semantic inconsistency, whose predictive value will be discussed later, the article by Olson et al., demonstrates that the tone of the discourse can also be important.

By using Linguistic Inquiry and Word Count, they determine that despite any significant differences in the count of emotionally charged terms, the tone of speech becomes more “negative” in CHR-P patients, particularly if their positive symptoms are high. This result perfectly represents the subtlety of language and the complex interactions between its various levels. Both in the clinical interview and classification criteria, the assessment of emotional states is an integral part of the diagnostic process. In clinical practice, these are spontaneously perceived and often identified through vocal expression, particularly in prosody, intonation, rhythm, or even intensity. To build on these results, it is interesting to remember that paralinguistic abnormalities linked to emotion (e.g., monotone voice, prosody flattening) (14–16) probably reflect affects in a complementary way to verbal content when detection of psychiatric disorders is at stake.

According predictive value, the study by Kim-Dufor et al., uses transcripts of free speech interviews as input to a machine learning model (XGBoost) (17) to automatically classify with 82% accuracy success patients into three categories: not at risk, at risk, and first psychotic episode. The authors also examine the respective contribution to the classification of linguistic markers in the transcribed speech and conclude that semantic coherence, frequency of pronoun “I,” and filled pauses help in predicting patient’s outcome. This approach reconciles algorithmic performance and clinical intelligibility, providing a more transparent “black box,” which constitutes an essential condition for the acceptability of AI tools in everyday psychiatric practice.

Finally, the relationships between the aforementioned linguistic anomalies and their neural correlates have also been studied in this topic. Applying the PRISMA method on 37 imaging studies, Alonso-Sánchez et al., explored the link between linguistic disturbances such as semantic coherence, maximal semantic coherence or disorganization of thoughts and brain alterations. Thus, whether patients are at ultra-high risk (UHR), have already had a first episode of psychosis (FEP) or present SSD, structural and functional modifications appear. These are mainly driven by differences in processing both in production or comprehension of speech when semantics is involved whether analyzed via NLP models or introduced employing specific experimental paradigms. Functional changes are also found related to disorders of encoding and/or word selection; two functions closely intertwined in the construction of a semantically coherent discourse. The pattern of visible changes also seems to extend from patients with FEP to those with schizophrenia, through UHR patients.

## Conclusion

The linguistic and cognitive disruptions highlighted throughout this Research Topic not only deepen our understanding of schizophrenia spectrum disorders but also pave the way toward the development of more faithful cognitive models of language processing, potentially surpassing existing connectionist frameworks (18).

Beyond the diagnostic domain, these AI-driven approaches hold great promise by automating language analysis to provide more objective, faster, and cost-effective evaluations than traditional clinical methods. Such tools could contribute to a psychiatry that is more precise, personalized, and predictive, capable of detecting subtle changes well before the onset of severe symptoms. This progress could also, one day, enable innovative applications like the emergence of a digital twin of the brain’s language functions, offering unprecedented insights into psychosis.

It is important to emphasize that artificial intelligence will never replace the clinician but rather become his most valuable ally, especially in the complex and inherently subjective field of mental health. Once known as a “language disease,” schizophrenia today finds in digital language processing an innovative tool for understanding and care, opening new horizons for both research and clinical practice.
